# Understanding the Spectrum of *SLC2A1*-Associated Disorders

**DOI:** 10.15844/pedneurbriefs-31-2-1

**Published:** 2017-02

**Authors:** Marytery Fajardo, Melissa L. Cirillo

**Affiliations:** 1 Division of Neurology, Ann & Robert H. Lurie Children’s Hospital of Chicago, Chicago, IL; and Departments of Pediatrics and Neurology, Northwestern University Feinberg School of Medicine, Chicago, IL

**Keywords:** *SLC2A1*, GLUT1, Epilepsy

## Abstract

Investigators from the Danish Epilepsy Center the frequency of *SLC2A1* mutations in a cohort of patients with different types of epilepsies.

Investigators from the Danish Epilepsy Center the frequency of *SLC2A1* mutations in a cohort of patients with different types of epilepsies. Based on recent reports describing *SLC2A1* mutations in patients with myoclonic astatic epilepsy (MAE), early onset absence epilepsy (EOAE), and genetic generalized epilepsies (GGEs), they aimed to replicate these findings in a cohort of European patients. They recruited 120 patients with MAE, 50 with absence epilepsy (not specifically early onset absence), as well as 37 patients with a variety of unspecified epilepsies, intellectual disability, and/or movement disorders. The investigators sequenced all exons and exon-intron boundaries using standard Sanger sequencing or high throughput next-generation sequencing. They found mutations in *SLC2A1* in six patients; five with absence epilepsy and one from the unspecified epilepsies, intellectual disability, and/or movement disorders group. No mutations were identified in patients with MAE. They found the mutation in question had been inherited in one of the patients, and that the mother also had absence seizures. Additionally, this patient and her mother had a microarray that indicated a maternally inherited 15q13.3 microdeletion. Four of the patients with *SLC2A1* mutations had classical absence epilepsy, one had epilepsy with myoclonic absences, and one had been diagnosed with focal epilepsy, though this patient had generalized discharges on EEG. Four of the six patients with *SLC2A1* mutations achieved seizure freedom with ketogenic or modified Atkins diets.

The investigators additionally sought to estimate the frequency of diagnosed glucose transporter type 1 deficiency syndrome (GLUT1 DS) in Denmark by identifying all diagnosed Danish patients and the number of live births between 2004 and 2011. They estimated that the 8-year frequency of GLUT1 DS was 1:83,245, which correlated with a previously reported incidence in Australia of 1:90,000 [1,2].

COMMENTARY. GLUT1 DS represents the phenotype historically associated with *SLC2A1* mutations. The classic form is associated with epileptic encephalopathy and complex movement disorders, whereas milder forms with primary movement disorders and other paroxysmal symptoms are also described [3]. The authors estimated the frequency of diagnosed GLUT1 DS in Denmark as 1:83,245, though many patients may remain undiagnosed. As genetic testing becomes more widespread, the phenotypic variation of *SLC2A1*-associated disorders continues to expand, and the cases reported here highlight clinical spectrum that can be seen [4]. Though none of their patients with MAE had *SLC2A1* mutations, the investigators discussed a previously reported cohort that identified *SLC2A1* mutations in four of 84 patients with MAE. These patients exhibited an MAE “plus” phenotype (associated movement disorders, motor and/or speech difficulties), which the authors hypothesized could be related to their mutation [5]. The investigators’ work adds to a growing body of data advancing our understanding of the correlation between different pathogenic variants and their clinical phenotypes and emphasizes the value of genetic testing in patients with generalized epilepsies and additional neurologic findings [4].
